# Beyond Gross Motor Function: The Association Between Fine Motor Skills and Cognitive Development in Treated SMA Type 1

**DOI:** 10.3390/jcm15207999

**Published:** 2026-10-15

**Authors:** Sofia Biagi, Livia Rinaldi, Maria Teresa Arnoldi, Claudia Dosi, Sofia Gandolfi, Ilaria Pedrinelli, Stefano Parravicini, Marta Viganò, Riccardo Zanin, Riccardo Masson

**Affiliations:** 1 Developmental Neurology Unit, Fondazione IRCCS Istituto Neurologico Carlo Besta, 20133 Milan, Italy; sofia.biagi@istituto-besta.it (S.B.); liviarinaldi.npi@gmail.com (L.R.); mariateresa.arnoldi@istituto-besta.it (M.T.A.); claudia.dosi@istituto-besta.it (C.D.); ilaria.pedrinelli@istituto-besta.it (I.P.); stefano.parravicini@istituto-besta.it (S.P.); marta.vigano@istituto-besta.it (M.V.); riccardo.zanin@istituto-besta.it (R.Z.); riccardo.masson@istituto-besta.it (R.M.); 2 Department of Innovation Medicine and Engineering, University of Verona, 37134 Verona, Italy

**Keywords:** spinal muscular atrophy type 1, BSID-III, neurodevelopment, fine motor skills, cognitive profile, embodied cognition

## Abstract

**Background:** Disease-modifying therapies (DMTs) have transformed the natural history of spinal muscular atrophy (SMA) type 1, revealing heterogeneous developmental trajectories and renewing interest in cognitive and language outcomes beyond gross motor function. Within an embodied cognition framework, sensorimotor experience, object manipulation, and interactions with the environment may shape early cognitive development. This study aimed to describe the cognitive, language, and motor development of treated children with SMA type 1 and to explore associations between gross motor abilities and neurodevelopmental outcomes. **Methods:** A retrospective observational study included 32 children aged 18–30 months with confirmed SMA type 1. Participants were assessed using the Children’s Hospital of Philadelphia Infant Test of Neuromuscular Disorders (CHOP-INTEND) at baseline (T0) and both CHOP-INTEND and the Bayley Scales of Infant and Toddler Development, Third Edition (BSID-III) at the time of neurodevelopmental assessment (T1). Associations among Fine Motor, Cognitive, Language, and Gross Motor scores were examined using Spearman’s correlations and multivariable rank-based regression. **Results:** Fine Motor scores showed inter-individual variability and were correlated with Cognitive (ρ = 0.647, *p* < 0.001) and Language (ρ = 0.753, *p* < 0.001) scores. The association between Fine Motor and Cognitive performance remained robust even after adjustment for gross motor function, assessed by CHOP-INTEND, indicating that fine motor ability is linked to cognitive outcome irrespective of overall motor severity. **Conclusions:** In our cohort, fine motor ability was associated with cognitive and language outcomes, regardless of gross motor severity. These findings suggest the important role of neurodevelopmental components, beyond gross motor function alone, supporting their systematic integration into the clinical characterization of children with treated SMA type 1.

## 1. Introduction

Spinal muscular atrophy (SMA) is an autosomal recessive neurodegenerative disease characterized by degeneration of spinal cord motor neurons. It results from homozygous disruption of the survival motor neuron 1 (*SMN1*) gene, which may occur through deletion, gene conversion, or mutation [1]. The homologous survival motor neuron 2 (*SMN2*) gene produces predominantly truncated, non-functional SMN protein but also generates a small amount of full-length functional SMN protein, which partially compensates for the loss of *SMN1*. The number of *SMN2* copies is closely associated with clinical severity, with a higher copy number generally correlating with a milder disease phenotype [2].

SMA has been traditionally categorized into the following types based on the highest motor milestone achieved and the age of symptom onset: SMA type 0 infants present with very severe hypotonia and require respiratory support from birth. These SMA infants cannot survive beyond 6 months. Type 1 (associated with usually one or two copies of *SMN2*) is characterized by severe hypotonia with onset during the first six months of life, leading to an inability to sit independently [2]; swallowing is compromised, and these infants require ventilatory support [3]. Type 2 (associated with two to three copies of *SMN2*) is characterized by symptom onset before 18 months of age, progressive proximal muscle weakness, and hypotonia. Patients are able to sit independently but are unable to achieve walking. Patients with SMA type 3 (associated with three to four copies of *SMN2*) develop symptoms after 18 months and are able to walk with difficulty. Type 4 (associated with more than four copies of *SMN2*): this phenotype is characterized by the onset of muscle weakness in adulthood. Motor impairment is usually mild, allowing patients to maintain independent ambulation, and respiratory and nutritional functions are preserved [2]. Table 1 summarizes the clinical characteristics of the different SMA subtypes.

The natural history of SMA type 1 has changed substantially in the past decade following the introduction of innovative pharmacological treatments. Currently, three main pharmacological treatments are available: nusinersen is an intrathecally administered *SMN2*-targeting antisense oligonucleotide and the first therapy for SMA approved by the US Food and Drug Administration (FDA) in 2016 [4]; onasemnogene abeparvovec is an intravenously administered adeno-associated virus vector-based gene-transfer therapy approved by FDA in 2019 [5]; risdiplam is the first oral medication approved for the treatment of SMA and targets *SMN2* to increase the production of viable SMN protein [6,7].

With the advent of disease-modifying therapies (DMTs), children with SMA now achieve functional abilities that differ markedly from those observed in the pre-treatment era and exhibit highly heterogeneous developmental trajectories. These advances have renewed interest in investigating cognitive development, particularly how enhanced motor function and richer interactions with the physical environment may shape developmental outcomes. Existing evidence on cognitive and language development in SMA remains limited and partly inconsistent. Early studies conducted before the DMTs era reported contradictory findings, with cognitive performance ranging from preserved to significantly impaired depending on the assessment tool and its dependence on motor responses [8,9]. In treated cohorts, data from developmental scales have repeatedly shown a dissociation between domains: language and learning trajectories tend to be comparatively more favorable than gross motor outcomes, which remain the primary driver of overall developmental delay even after treatment [10].

This discrepancy across findings reflects the lack of standardized assessment protocols and the pervasive influence of motor-dependent test items, which makes it difficult to distinguish genuine neurodevelopmental disorders from the indirect effects of motor impairment [11].

In milder SMA phenotypes, cognitive functioning has traditionally been considered unaffected, with early studies reporting normal intelligence and language development compared to healthy peers [11,12]. More recent investigations, however, have identified domain-specific vulnerabilities—such as reduced working memory and perceptual reasoning—despite globally preserved IQ [13].

In recent years, theories of embodied cognition have progressively reshaped our understanding of cognitive development, emphasizing that learning emerges from the dynamic interaction between brain, body, and environment. According to this perspective, sensorimotor experiences, object manipulation, and active exploration of the environment are fundamental components of cognitive development. These experiences contribute to the formation of mental representations and play a crucial role in shaping the organization and functioning of cognitive processes [14]. In children with severe motor impairment, opportunities for active interaction with the environment are inevitably reduced, making it particularly relevant to understand whether and how functional limitations may influence developmental outcomes.

For many years, researchers focused on motor and respiratory outcomes of children with SMA, while cognitive development and communication skills have been investigated only in a limited number of studies, often involving small cohorts and heterogeneous assessment methods [15]. The aim of the present study was to describe the cognitive, language, and motor developmental profile of a cohort of children with SMA type 1, exploring the relationship between developmental domains and gross motor function in order to identify potential associations between gross and fine motor function and neurodevelopmental outcomes.

## 2. Materials and Methods

This is an observational retrospective study including patients with SMA type 1 evaluated at the Developmental Neurology Unit of Istituto Neurologico Carlo Besta in Milan, from January 2019 to December 2025. All procedures were performed in accordance with Good Clinical Practice guidelines and the Declaration of Helsinki. This study was approved by the local ethics committee.

As part of the clinical routine at our hospital, children with early-onset neuromuscular disease undergo a neurodevelopmental assessment usually starting from the age of 12–24 months. For SMA patients, the Bayley Scales of Infant and Toddler Development, Third Edition (BSID-III) was selected as the preferred tool due to its ability to separately analyze gross motor, fine motor, and cognitive development [16].

In the present study, we enrolled patients with a genetic diagnosis of SMA 5q and a clinical profile of SMA type 1. According to the standard of care, all the included children had received a DMT after symptom onset. An additional inclusion criterion was to have at least one assessment using the BSID-III between the ages of 18 and 30 months, as this age range is conventionally considered the best window to look at the early developmental abilities.

When more tests were available, we selected the one closest to the age of 24 months.

Exclusion criteria were clinical diagnosis of other forms of SMA, different from type 1; presymptomatic SMA at the time of treatment initiation; neurodevelopmental assessment with tools other than the BSID-III or having the test before 18 months or after 30 months of age; not having received any DMT.

### 2.1. Neurodevelopmental Assessment

Neurodevelopmental assessment was performed using BSID-III, administered according to the standardized procedures described in the test manual. Composite scores for the *Cognitive*, *Language*, and *Motor* scales were collected. In addition, the scaled scores of the *Language* subscales (*Receptive* and *Expressive*) and *Motor* subscales (*Fine Motor* and *Gross Motor*) were recorded and included in the analyses. BSID-III was administered by two trained psychologists (S.B., S.M.) with experience in assessing children affected by neuromuscular disease.

### 2.2. Motor Function Assessment

Gross motor abilities were assessed through the Children’s Hospital of Philadelphia Infant Test of Neuromuscular Disorders (CHOP-INTEND) [17], administered by two highly trained motor therapists (R.Z., M.T.A.). CHOP-INTEND is a routine assessment for patients with SMA type 1. We included the baseline CHOP-INTEND score in this study, obtained for each patient before the DMT initiation (CHOP-T0), and the score achieved at the time of neurodevelopmental assessment (CHOP-T1).

For each participant, the following clinical data were collected:

- Sex;
- *SMN2* copy number;
- Age at symptom onset;
- Age at DMT initiation;
- Delay between age at symptom onset and DMT initiation;
- Type of DMT received;
- Age at neurodevelopmental assessment;
- BSID-III scores for *Cognitive*, *Language* and *Motor* scales;
- CHOP-T0 and CHOP-T1 scores.

We analyzed the associations of cognitive development (Cognitive Composite score) and fine motor skills (Fine Motor scaled score) with gross motor abilities (CHOP-INTEND score) and the other demographic and clinical features.

Furthermore, we investigated potential differences in the same parameters by dividing the SMA type 1 population into two subgroups according to their Cognitive Composite scores: below the normal range (Cognitive score < 85) and within the average range (Cognitive score ≥ 85).

### 2.3. Statistical Analysis

Statistical analyses were performed using SPSS Statistics (version 30.0, IBM SPSS Inc., Armonk, NJ, USA). Normality of continuous variables was assessed using the Shapiro–Wilk test. Missing data were not imputed. Analyses were conducted on available data: correlations were computed using pairwise deletion, and regression models and non-parametric tests were fitted on patients with complete data for the variables involved. The number of patients with missing data for each variable, and the number of observations included in each analysis, are reported in Table 2, Table 3 and Table 4. When continuous variables were non-normally distributed, data were presented as median (interquartile range [IQR]). Categorical variables were expressed as frequencies (%). Non-normally distributed variables were analyzed using the Mann–Whitney U test to assess the difference between two subgroups and using the Wilcoxon test to assess the change in gross motor function between baseline and the time of assessment. Bivariate associations were assessed using Spearman’s rank correlation coefficients, with 95% confidence intervals computed using the Bonett–Wright method [18]. The strength of the correlations was interpreted according to the classification proposed by Schober, Boer, and Schwarte [19]. To investigate whether the observed associations were independent of other variables, multivariable analyses were performed using rank-transformed variables, following the approach proposed by Conover and Iman [20]. Specifically, separate multiple linear regression models were fitted to assess whether the associations between Fine Motor score and, respectively, cognitive and language performance remained significant after adjustment for gross motor ability and treatment delay. For each outcome (Cognitive score and Language score), three models were fitted using rank-transformed variables, with the ranked outcome as the dependent variable and the ranked Fine Motor score as the main predictor; the three models differed in the single covariate included, namely the ranked CHOP-T0 score, the ranked CHOP-T1 score, or the delay between symptom onset and DMT administration, respectively. Model assumptions were evaluated by inspection of standardized residuals and normal probability plots. Multicollinearity was assessed using tolerance values and variance inflation factors (VIFs), with VIF > 5 considered indicative of multicollinearity.

To account for multiple comparisons, *p*-values from the correlation analyses were adjusted using the Bonferroni procedure, controlling the family-wise error rate at 5%. Bonferroni correction was applied specifically to the correlation analyses, which represented a family of exploratory bivariate associations tested across multiple variables: the Cognitive composite, Fine Motor scaled score and Language composite score, CHOP-INTEND scores (T0 and T1), age at symptoms onset, age at treatment initiation, and treatment delay, for a total of 18 correlations, corresponding to an adjusted significance threshold of α = 0.05/18 = 0.0028. *p*-values reported in the text are unadjusted (raw); Bonferroni-adjusted *p*-values are reported in Table S2. Confidence intervals were not adjusted for multiple comparisons. Subgroups comparisons were conducted as exploratory analyses aimed at characterizing clinical and developmental differences between groups defined according to a normative threshold on the Cognitive Composite score (<85 vs. ≥85); therefore, *p*-values were not adjusted for multiplicity and were interpreted as exploratory rather than confirmatory findings.

## 3. Results

**Figure 1:**
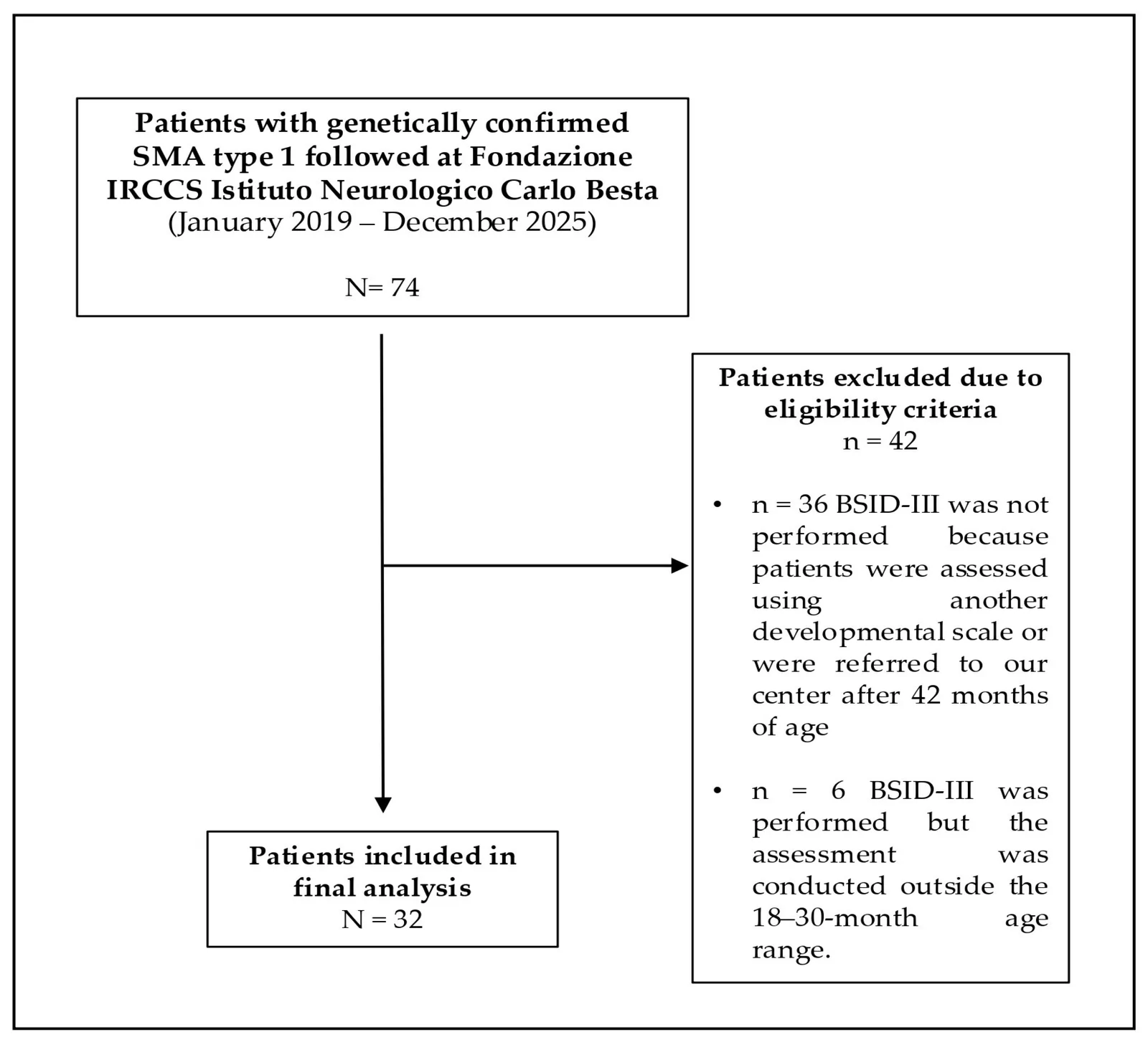
Patients’ eligibility flowchart.

Between January 2019 and December 2025, 74 children with genetically confirmed SMA type 1 were assessed at our center. Of these, 32 patients fulfilled the eligibility criteria and were included in this study (Figure 1).

Demographic and clinical characteristics of the study population, including motor function as assessed by CHOP-T0 and CHOP-T1, are summarized in Table 2. At both baseline and at the time of the BSID assessment, CHOP-INTEND scores showed a wide range in the study population, with a significant increase from T0 (median = 29) to T1 (median = 51.5), W = 435, *p* < 0.001. Regarding the *SMN2* copy number, 4 children had 3 *SMN2* copies and 28 had 2 *SMN2* copies. The clinical characteristics of the four children with three *SMN2* copies are reported in Table S1 in the Supplementary Materials.

### 3.1. Neurodevelopmental Profiles

The results of the neurodevelopmental assessment (Cognitive, Language, and Motor scores on the BSID-III) for the entire cohort are reported in Table 3. Overall, children with SMA type 1 exhibited a heterogeneous developmental profile across the different BSID-III domains, with Cognitive and Language performances better preserved than Motor abilities. Within the motor domain, the Gross Motor scaled score was markedly reduced across the cohort, consistent with the severe motor phenotype typically associated with SMA type 1 (Gross Motor score: median = 1). In contrast, fine motor abilities showed greater variability (Fine Motor score: median = 7, range 2–16).

### 3.2. Associations Between Neurodevelopmental Outcomes, Motor Function and Other Clinical Parameters

In the whole cohort, the *Fine Motor* score was associated with both the *Cognitive Composite* score (ρ = 0.647, *p* < 0.001) and the *Language Composite* score (ρ = 0.753, *p* < 0.001). The *Cognitive* and *Language* scores were also correlated (ρ = 0.761, *p* < 0.001). All three correlations remained statistically significant after Bonferroni correction for multiple comparisons (adjusted α = 0.0028).

As correlations between the Gross *Motor* scaled score and the other parameters could not be assessed, the CHOP-INTEND score was used as a proxy measure of gross motor function in patients with SMA. Neither the *Cognitive Composite* score nor the *Fine Motor* score was significantly associated with either CHOP-T0 or CHOP-T1. Specifically, associations between the *Cognitive Composite* score and the CHOP-T0 (*p* = 0.195) or CHOP-T1 (*p* = 0.073), and between the *Fine Motor* score and CHOP-T0 (*p* = 0.164), did not reach statistical significance. Although the *Fine Motor* score was significantly correlated with CHOP-T1 before correction (*p* = 0.039), this association did not remain statistically significant after Bonferroni correction for multiple comparisons. Age at symptom onset, age at DMT initiation, and treatment delay were not associated with either *Fine Motor* (*p* = 0.126, 0.331, and 0.764, respectively) or *Cognitive* scores (*p* = 0.969, 0.810, and 0.602, respectively). See Table S2 in the Supplementary Materials for all Confidence Intervals (CIs) and adjusted *p*-values.

Multivariable analyses were performed using rank-transformed variables to test the robustness of the association between *Fine Motor* score and, respectively, cognitive and language performance, after adjustment for gross motor function and treatment delay.

When ranked CHOP-INTEND at baseline was included in the model together with *Fine Motor* score, the regressions were statistically significant for both outcomes (Cognitive: F(2,25) = 9.02, *p* = 0.001; R^2^ = 0.419; Language: F(2,25) = 19.11, *p* < 0.001, R^2^ = 0.605), with *Fine Motor* score remaining associated with cognitive outcome (β = 0.629, *p* < 0.001) and language outcome (β = 0.719, *p* < 0.001), whereas CHOP-T0 was not a significant predictor for either outcome (Cognitive: β = 0.059, *p* = 0.715; Language: β = 0.158, *p* = 0.237). No relevant multicollinearity was observed (VIF = 1.08 and 1.079, respectively).

Similarly, when ranked CHOP-INTEND at the time of assessment was included as covariate, both models remained significant (Cognitive: F(2,28) = 10.10, *p* < 0.001; R^2^ = 0.419; Language: F(2,28) = 18.33, *p* < 0.001, R^2^ = 0.567), with *Fine Motor* score again associated with cognitive (β = 0.639, *p* < 0.001) and language outcome (β = 0.725, *p* < 0.001), whereas CHOP-T1 was not significant for either outcome (Cognitive: β = 0.021, *p* = 0.892, VIF = 1.16; Language: β = 0.069, *p* = 0.612, VIF = 1.162).

Finally, adjusting for treatment delay yielded consistent results for both outcomes (Cognitive: F(2,28) = 10.11, *p* < 0.001, R^2^ = 0.419; Language: F(2,28) = 20.60, *p* < 0.001, R^2^ = 0.595): *Fine Motor* score remained associated with cognitive (β = 0.651, *p* < 0.001) and language performance (β = 0.741, *p* < 0.001), whereas treatment delay was not a significant predictor for either outcome (Cognitive: β = −0.026, *p* = 0.859, VIF = 1.00; Language: β = −0.180, *p* = 0.145, VIF = 1.003).

Overall, these analyses indicate that the association between *Fine Motor* and both *Cognitive* and *Language* performance is robust and independent of gross motor function (both at baseline and at the time of assessment) and of treatment delay. Model assumptions were verified by inspection of standardized residuals and normal probability plots for all six regression models (Table S3 in the Supplementary Materials).

### 3.3. Subgroup Analysis

The whole population was divided into two subgroups based on *Cognitive* score: 14 children with scores below the average range (*Cognitive* score < 85) and 18 children within the average range (*Cognitive* score ≥ 85) (Table 4). Children with below-average *Cognitive* score showed lower *Language* and *Fine Motor* scores. No significant differences were observed for the other clinical or demographic characteristics, including age at symptom onset, age at DMT initiation, treatment delay, and age at assessment with BSID-III. CHOP-T1 scores did not differ significantly between the two subgroups, whereas CHOP-T0 scores were lower in children with below-average Cognitive scores, although this difference should be interpreted with caution given the number of comparisons performed and the reduced sample size for this measure.

## 4. Discussion

DMTs have profoundly transformed the natural history of SMA: children with SMA type 1 now reach developmental milestones and achieve functional abilities previously unattainable, resulting in markedly heterogeneous developmental trajectories. Understanding how other developmental domains, such as cognition and language, evolve and how they relate to residual motor abilities has therefore become essential not only for a more complete characterization of disease severity but also for guiding rehabilitation planning and school inclusion strategies tailored to each child’s individual developmental profile. Nevertheless, although the characterization of cognitive development is now considered clinically relevant, the systematic collection of data remains limited.

We decided to focus our analysis on children with SMA type 1 assessed between 18 and 30 months of age, as this developmental window is considered particularly informative for examining early cognitive and motor abilities. During this period, manual exploration, object manipulation, and active interaction with the surrounding environment play a foundational role in supporting cognitive development, providing the sensorimotor experiences on which early representational and cognitive processes are built [21,22]. At the same time, within this age range, the fine motor demands of the BSID-III are still accessible to children with SMA type 1, allowing the scales to be administered in their original standardized form, without adapting any of the tasks. At older ages, by contrast, BSID-III tasks require progressively finer and more precise movements while also assessing the speed of motor execution, which would penalize these children. Restricting the assessment to this window therefore reduces the risk that motor limitations contaminate both the estimate of cognitive ability and the measurement of fine motor performance. Examining the relationship between motor and cognitive functions requires a tool capable of capturing multiple developmental domains within a single, standardized assessment. We selected the BSID-III to address this need, as it provides a simultaneous assessment of cognitive, language, and both fine and gross motor development, allowing the relationships between these domains to be examined directly rather than through the combination of different assessment tools [16].

Our study indicates that, unlike gross motor function, which is often severely compromised, fine motor abilities may show greater inter-individual variability in this population. Understanding whether this variability is associated with cognitive and language development could provide new insights into developmental mechanisms in children with SMA and help identify, at an early stage, those who may benefit from targeted rehabilitative interventions.

As the BSID-III was developed and normed for the general pediatric population, its *Gross Motor* subscale is largely based on the achievement of ambulatory milestones. Consequently, all children with SMA type 1 score at the floor of this subscale, which fails to capture meaningful variability in global motor function. For this reason, gross motor performance was also assessed using the CHOP-INTEND, a scale specifically developed and validated for infants with SMA type 1 [17]. In contrast, the *Fine Motor* subscale retains sufficient range in this population to reflect individual differences and was therefore used to characterize fine motor abilities.

Children in our cohort showed cognitive and language scores below normative values, with median composite scores of 85 (at the lower limit of the average range) and 80, respectively, and profound impairment of gross motor function, with all children scoring at the floor of the BSID-III *Gross Motor* subscale, and preserved inter-individual variability in fine motor performance. In the whole cohort, fine motor performance was positively associated with both *Cognitive* and *Language* scores; *Cognitive* and *Language* scores were also strongly associated. These findings suggest that fine motor abilities, cognition, and language tended to vary together, indicating that these domains are developmentally interrelated in children with treated SMA type 1. The strong association between language and cognitive scores is consistent with the literature [23]: in children of this age, language and cognition are closely intertwined: receptive language, in particular, relies heavily on the same emerging representational and comprehension abilities that support cognitive performance; furthermore, many language items at this developmental stage—understanding simple requests, recognizing objects, following simple directions—draw on cognitive and purely linguistic resources. The parallel behavior of the two domains in our cohort is therefore consistent with the developmental interdependence between early language and cognition, and suggests that adequate cognitive functioning, together with a stimulating and responsive environment, may play an important role in supporting language acquisition in children with SMA type 1, who often face reduced opportunities for autonomous exploration and environmental interaction. The reduced language scores we observed may reflect, at least in part, the broader cognitive profile of these children rather than a language-specific impairment.

The most relevant finding, however, emerged when the relationship between *Cognitive* score and *Fine Motor* score was examined in relation to overall motor function. The significant association observed between fine motor performance and cognitive outcome remained essentially unchanged after adjustment for gross motor function, suggesting that the relationship between these two domains is not simply explained by differences in gross motor ability. Conversely, no significant association was observed between cognitive performance and CHOP-INTEND scores in the whole cohort. This apparent discrepancy between the absence of this association and the lower baseline CHOP-INTEND scores observed descriptively in the below-average *Cognitive* subgroup should be interpreted cautiously due to the limited sample size.

Overall, this finding may indicate that cognitive and gross motor outcomes capture partially distinct aspects of the disease. However, the absence of a direct association between CHOP-INTEND scores and cognitive performance does not necessarily exclude a contribution of overall disease severity to cognitive development. Indeed, if disease severity represents an underlying construct influencing both motor and cognitive outcomes, CHOP-INTEND score may capture only part of this construct, particularly its motor component.

Therefore, the observed pattern of results—a robust association between *Fine Motor* and *Cognitive* scores, together with the absence of a significant association between CHOP-INTEND score and cognitive performance—may be consistent with the hypothesis that cognitive development is influenced by factors beyond gross motor impairment, potentially including overall disease severity. This hypothesis is consistent with observations in other aspects of the disease, such as the previously described discrepancy between motor outcomes and swallowing function in stronger patients with SMA type 1 [24].

At the same time, these findings can be considered within the framework of embodied cognition, which posits that cognitive development is grounded in sensorimotor interactions between the child and the environment. From this perspective, during the earliest stages of development, manual exploration and object manipulation support cognitive development by generating the perceptual and action-based experiences on which emerging representations are built. This framework provides a coherent account of the specificity of the association we observed: fine motor ability—which underlies reaching, grasping, and the active exploration of objects—was associated with cognitive performance, whereas global gross motor function was not. A complementary, non-mutually exclusive explanation is that fine motor and cognitive abilities share common underlying processes and neural substrates. Motor and cognitive development have been shown to be closely interrelated, with partly overlapping neural circuits, particularly involving the cerebellum and prefrontal cortex [25]. Consistently, a recent meta-analysis in typically developing children and adolescents reported an association between fine motor skills and cognitive development, supporting the view that the two domains rely on shared processes [26].

To our knowledge, this is the first study to investigate the specific relationship between fine motor skills, cognition, and gross motor development in children with treated SMA type 1. Two recent studies have examined the neurodevelopmental profile of presymptomatic patients identified through newborn screening using the BSID-III and both suggest the important role of neurodevelopmental domains, beyond gross motor function alone, as key components that help define overall disease severity in SMA [27,28]. Our findings extend these observations to a clinically diagnosed, symptomatic cohort. Taken together, these findings reinforce the need to systematically monitor neurodevelopmental domains in children with treated SMA, as cognitive, language, and fine motor outcomes may themselves carry important information on overall disease severity and should therefore be integrated into the clinical characterization of these patients.

These hypotheses remain to be tested in future studies. The main limitations of our study are its retrospective design, and the limited number of patients enrolled, which precluded analysis of DMT-specific effects. Further limitation concerns the fact that some *Cognitive* items involve reaching or object manipulation; shared task demands may therefore partly contribute to the observed correlation. However, the two scales differ in the specific construct assessed: *Cognitive* items are scored primarily on whether the child achieves the task goal, without penalizing execution time, movement quality, or alternative strategies, whereas the *Fine Motor* scale specifically evaluates precision, grasp pattern, and speed of execution. This distinction limits the overlap in task demands. On the other hand, the characteristics of our cohort broadly reflect those of patients with SMA type 1 currently treated in clinical practice, as indicated by the young age at treatment initiation and the wide range of baseline CHOP-INTEND scores, reflecting the inclusion of patients with different levels of disease severity. Furthermore, the assessment methodology was highly consistent across the different assessments, including the evaluators involved. Longitudinal, multicenter studies with larger cohorts are needed to confirm and extend these findings.

## 5. Conclusions

In our cohort of 32 children with treated SMA type 1, fine motor ability was associated with cognitive and language outcomes, regardless of gross motor severity measured by CHOP-INTEND. These findings support the systematic integration of fine motor, cognitive, and language assessment into the clinical characterization of children with treated SMA type 1 and raise the hypothesis that neurodevelopmental components beyond gross motor function alone may contribute to a broader picture of disease impact.

## Figures and Tables

**Table 1:** Clinical classification of SMA subtypes.

Type	Age of Onset	Able to Sit	Able to Stand	Able to Walk	Predicted SMN2 Copy Number	Requires Respiratory Support at Birth	Life Expectancy Without DMTs
0	Prenatal	No	No	No	1	Yes	<6 months
1	<6 months	No	No	No	1–2–3	No	<2 years
2	6–18 months	Yes	No	No	2–3	No	10–40 years
3	>18 months	Yes	Yes	Assisted	3–4	No	Adult
4	>18 years	Yes	Yes	Yes	>4	No	Adult

DMTs = disease-modifying therapies.

**Table 2:** Demographic and clinical characteristics of the study population (N = 32).

**Demographic characteristics**	
**Sex, n (%)**	
Female	18 (56.3)
Male	14 (43.7)
**Age at assessment, months, median (IQR)**	23.0 (6.8)
**Clinical characteristics**	
***SMN2* copy number, n (%)**	
2 *SMN2* copies	28 (87.5)
3 *SMN2* copies	4 (12.5)
**First-line DMT, n (%)**	
onasemnogene abeparvovec	15 (46.9)
risdiplam	11 (34.4)
nusinersen	6 (18.7)
**Age at symptom onset, months, median (IQR)**	1.6 (2.0)
**Age at DMT initiation, months, median (IQR)**	4.4 (3.0)
**Treatment delay, months, median (IQR)**	2.8 (2.7)
**CHOP-INTEND at T0, median (IQR) ^1^**	29.0 (12.0)
**CHOP-INTEND at T1, median (IQR)**	51.5 (10.0)

^1^ CHOP-INTEND data at T0 were available for 29 of 32 patients.

**Table 3:** BSID-III neurodevelopmental outcomes in the study cohort.

Domain	Normative Reference Values, Median (IQR)	Median (IQR)
*Cognitive* Composite score	100.0 (20.0)	85.0 (22.5)
*Cognitive* scaled score	10.0 (4.0)	7.0 (4.0)
*Language* Composite score	100.0 (20.0)	80.0 (38.5)
*Receptive Communication* scaled score	10.0 (4.0)	7.0 (7.0)
*Expressive Communication* scaled score	10.0 (4.0)	6.0 (6.0)
*Motor* Composite score	100.0 (20.0)	61.0 (21.3)
*Fine Motor* scaled score ^1^	10.0 (4.0)	7.0 (7.0)
*Gross Motor* scaled score	10.0 (4.0)	1.0 (0.0)

^1^ Fine motor data were available for 31 of 32 children. The cognitive scores of the 3-SMN2-copy subgroup were equally distributed between the two subgroups based on cognitive performance: two participants had a Cognitive score < 85, and two had a Cognitive Score ≥ 85.

**Table 4:** Clinical and developmental measures in children with below-average versus average cognitive scores (cognitive composite < 85 vs. ≥85).

Demographic and Clinical Characteristics	Cognitive Score < 85, Median (IQR)	Cognitive Score ≥ 85, Median (IQR)	U	r_rb_	*p*-Value
n	14	18			
Age at symptom onset, months	1.5 (1.3)	1.9 (2.0)	145	0.15	0.464
Age at DMT initiation, months	5.0 (3.6)	4.1 (2.8)	122	0.03	0.879
Treatment delay, months	3.3 (2.6)	2.6 (2.4)	103	0.18	0.391
Age at assessment, months	23.5 (4.8)	23.0 (8.3)	114.5	0.09	0.661
Language Composite score	57.0 (20.5)	92.5 (16.3)	235	0.87	<0.001 **
Fine motor scaled score ^1^	3.0 (4.0)	9.0 (4.3)	205.5	0.73	<0.001 **
CHOP-INTEND score at T0 ^2^	23.5 (10.0)	31.0 (9.0)	149	0.42	0.037 *
CHOP-INTEND score at T1	50.5 (10.0)	52.5 (12.0)	160	0.27	0.196

^1^ Fine motor data were available for 13 of 14 children with Cognitive Composite scores below 85 and for the totality of children with Cognitive Composite scores equal to or above 85. ^2^ CHOP-INTEND scores at T0 were available for 12 of 14 children with Cognitive Composite scores below 85 and for 17 of 18 children with Cognitive Composite scores equal to or above 85. *: statistically significant with *p* < 0.05; **: statistically significant result with *p* < 0.01; r**_rb_**: Rank-Biserial Correlation Coefficient; U: Mann-Whitney U statistic Statistical significance was set at *p* < 0.05.

## Data Availability

The original contributions presented in this study are included in this article; further inquiries can be directed to the corresponding author.

## Supplementary Materials

The following supporting information can be downloaded at https://www.mdpi.com/article/10.3390/jcm15207999/s1, Table S1: Comparison of clinical characteristics between the 3-SMN2-copy subgroup and the whole cohort; Table S2: Details of correlations between neurodevelopmental outcomes and clinical characteristics; Table S3: Regression models examining associations between neurodevelopmental outcomes and clinical characteristics.

## Abbreviations

The following abbreviations are used in this manuscript:

BSID-III	Bayley Scales of Infant and Toddler Development, Third Edition
CHOP-INTEND	Children’s Hospital of Philadelphia Infant Test of Neuromuscular Disorders
DMT	Disease-modifying therapy
FDA	Food and Drug Administration
IQR	Interquartile range
IRCCS	Istituto di Ricovero e Cura a Carattere Scientifico
SMA	Spinal muscular atrophy
SMN1	Survival motor neuron 1
SMN2	Survival motor neuron 2
T0	Baseline (pre-treatment) assessment time point
T1	Time of neurodevelopmental assessment
VIF	Variance inflation factor
