# Supplementary material for: Beyond Gross Motor Function: The Association Between Fine Motor Skills and Cognitive Development in Treated SMA Type 1
Source: J Clin Med. 2026 Oct 15;15(20):7999. doi: 10.3390/jcm15207999 (PMC13705689; doi:10.3390/jcm15207999)
Supplement: Supplementary file 1 [file jcm-15-07999-s001.zip › jcm-4554824-supplementary.pdf]

Table S1 Comparison of clinical characteristics between the 3-*SMN2*-copy subgroup and the whole cohort

**Clinical characteristics, median (IQR)**	**Children with 3 *SMN2* copies**	**Whole cohort**
Symptom onset, months	5.5 (1.8)	1.6 (2.0)
Age at DMT initiation, months	8.9 (3.2)	4.4 (3.0)
Treatment delay, months	4.4 (2.9)	2.8 (2.7)
Age at assessment, months	21.5 (9.0)	23.0 (6.8)
Cognitive Composite score	80.0 (13.8)	85.0 (22.5)
Language Composite score	91.0 (33.8)	80.0 (38.5)
Fine Motor scalar score	8.5 (4.0)	7.0 (7.0)
CHOP at T0	36.0 (NA)	29.0 (12.0)
CHOP at T1	50.5 (8.0)	51.5 (10.0)

CHOP = Children's Hospital of Philadelphia Infant Test of Neuromuscular Disorders; DMT = disease-modifying therapy; NA = Not Available due to the limited number of this subgroup; T0 = baseline assessment; T1 = follow-up assessment. The cognitive scores of the 3-SMN2-copy subgroup were equally distributed between the two subgroups based on cognitive performance: two participants had a Cognitive Score <85, and two had a Cognitive Score ≥85.

Table S2. Details of correlations between neurodevelopmental outcomes and clinical characteristics

	r_s	raw p-value	adjusted p-value	95% CI	
				inf	inf
Cognitive composite – Fine motor	0.647	< 0.001**	< 0.001**	0.348	0.827
Cognitive composite – Language composite	0.761	< 0.001**	< 0.001**	0.527	0.888
Cognitive composite – CHOP-T0	0.248	0.195	1.000	-0.136	0.567
Cognitive composite – CHOP-T1	0.321	0.073	1.000	-0.040	0.608
Cognitive composite – Symptom onset	0.007	0.969	1.000	-0.342	0.355

Cognitive composite – Age at treatment initiation	-0.044	0.810	1.000	-0.387	0.309
Cognitive composite – Treatment delay	-0.096	0.602	1.000	-0.431	0.263
Fine motor – Language composite	0.753	< 0.001**	0.002**	0.508	0.885
Fine motor – CHOP-T0	0.270	0.164	1.000	-0.121	0.589
Fine motor – CHOP-T1	0.373	0.039	0.702	0.009	0.650
Fine motor – Symptom onset	0.281	0.126	1.000	-0.089	0.582
Fine motor – Age at treatment initiation	0.181	0.331	1.000	-0.189	0.505
Fine motor – Treatment delay	-0.056	0.764	1.000	-0.403	0.305
Language composite – CHOP-T0	0.373	0.047	0.846	-0.006	0.658
Language composite – CHOP-T1	0.395	0.025	0.450	0.040	0.662
Language composite – Symptom onset	0.072	0.695	1.000	-0.284	0.411
Language composite – Age at treatment initiation	-0.077	0.677	1.000	-0.415	0.280
Language composite – Treatment delay	-0.248	0.171	1.000	-0.553	0.116

r_s = Spearman's rank correlation coefficient; CI = confidence interval; p adj = Bonferroni-adjusted p-value (m = 18; adjusted α = 0.0028). 95% CIs were computed using the Fisher z-transformation with the standard error proposed by Bonett and Wright and are not adjusted for multiple comparisons. ** indicates statistical significance after Bonferroni adjustment.

Table S3 Regression models examining associations between neurodevelopmental outcomes and clinical characteristics

Outcome	Covariate	N	F (df1, df2)	p	R^2	Adjusted R^2	Fine Motor B [95% CI]	Fine Motor *p*	Covariate B [95% CI]	Covariate p	VIF

Cognitive	CHOP-T0	28	F(2,25)=9.02	0.001	0.419	0.373	0.613 [0.295, 0.930]	< 0.001	0.061 [−0.280, 0.403]	0.715	1.079
Cognitive	CHOP-T1	31	F(2,28)=10.10	< 0.001	0.419	0.378	0.644 [0.324, 0.965]	< 0.001	0.021 [−0.298, 0.341]	0.892	1.162
Cognitive	Delay	31	F(2,28)=10.11	< 0.001	0.419	0.378	0.651 [0.353, 0.949]	< 0.001	−0.025 [−0.311, 0.261]	0.859	1.003
Language	CHOP-T0	28	F(2,25)=19.11	< 0.001	0.605	0.573	0.711 [0.445, 0.976]	< 0.001	0.168 [−0.118, 0.454]	0.237	1.079
Language	CHOP-T1	31	F(2,28)=18.33	< 0.001	0.567	0.536	0.733 [0.455, 1.010]	< 0.001	0.069 [−0.207, 0.346]	0.612	1.162
Language	Delay	31	F(2,28)=20.60	< 0.001	0.595	0.566	0.750 [0.500, 0.999]	< 0.001	−0.175 [−0.414, 0.064]	0.145	1.003

B = regression coefficient; CHOP = Children's Hospital of Philadelphia Infant Test of Neuromuscular Disorders; CI = confidence interval; VIF = variance inflation factor; T0 = baseline assessment; T1 = follow-up assessment.
